# The Regulation of ZIP8 by Dietary Manganese in Mice

**DOI:** 10.3390/ijms24065962

**Published:** 2023-03-22

**Authors:** Suetmui Yu, Ningning Zhao

**Affiliations:** School of Nutritional Sciences and Wellness, The University of Arizona, Tucson, AZ 85721, USA

**Keywords:** ZIP8, *SLC39A8*, manganese, liver, nutrient metabolism

## Abstract

ZIP8 is a newly identified manganese transporter. A lack of functional ZIP8 results in severe manganese deficiency in both humans and mice, indicating that ZIP8 plays a crucial role in maintaining body manganese homeostasis. Despite a well-acknowledged connection between ZIP8 and manganese metabolism, how ZIP8 is regulated under high-manganese conditions remains unclear. The primary goal of this study was to examine the regulation of ZIP8 by high-manganese intake. We used both neonatal and adult mouse models in which mice were supplied with dietary sources containing either a normal or a high level of manganese. We discovered that high-manganese intake caused a reduction in liver ZIP8 protein in young mice. Since a decrease in hepatic ZIP8 leads to reduced manganese reabsorption from the bile, our study identified a novel mechanism for the regulation of manganese homeostasis under high-manganese conditions: high dietary manganese intake results in a decrease in ZIP8 in the liver, which in turn decreases the reabsorption of manganese from the bile to prevent manganese overload in the liver. Interestingly, we found that a high-manganese diet did not cause a decrease in hepatic ZIP8 in adult animals. To determine the potential reason for this age-dependent variation, we compared the expressions of liver ZIP8 in 3-week-old and 12-week-old mice. We found that liver ZIP8 protein content in 12-week-old mice decreases when compared with that of 3-week-old mice under normal conditions. Overall, results from this study provide novel insights to facilitate the understanding of ZIP8’s function in regulating manganese metabolism.

## 1. Introduction

The systemic control of manganese homeostasis largely relies on the gastrointestinal tract and the liver—the intestine controls dietary manganese absorption, while the liver clears manganese through hepatobiliary excretion [1,2,3]. At the cellular level, the movement of manganese into or out of cells is mediated by plasma membrane metal transport proteins, including the newly identified manganese transporter, ZIP8. ZIP8 is ubiquitously expressed throughout the body and can mediate the cellular influx of several cations, such as manganese (Mn^2+^), zinc (Zn^2+^), and iron (Fe^2+^) [4,5,6]. The significance of ZIP8 in manganese metabolism was demonstrated in patients having loss-of-function mutations in the *ZIP8* (*SLC39A8*) gene. These patients displayed severe manganese deficiency and exhibited developmental delay, dystonia, and brain atrophy [7,8,9].

To examine the physiological functions of ZIP8, several studies utilized animal models. For example, one study found that the conventional knockout of *Zip8* causes embryo or neonatal lethality in mice [10]. This study concluded that ZIP8 was critical for embryonic organogenesis and hematopoiesis in utero. To further investigate ZIP8 function, another study established both tamoxifen-inducible global *Zip8* knockout (*Zip8*-KO) and liver-specific *Zip8* knockout (*Zip8*-LSKO) mouse strains [11]. Both strains developed manganese deficiency, which is consistent with the clinical observations in human patients carrying loss-of-function mutations in *ZIP8* [7,9]. Both *Zip8*-KO and *Zip8*-LSKO mice had significantly decreased manganese in the liver, kidney, brain, and heart compared with the wild-type controls [11]. The fact that *Zip8*-LSKO mice developed decreased manganese in the whole blood with ZIP8 inactivation only in the liver suggests that hepatic ZIP8 plays a significant role in regulating the whole-body manganese homeostasis [11]. Indeed, when *Zip8*-LSKO mice were injected with ZIP8-expressing AAV driven by a liver-specific promoter to restore hepatic ZIP8, these mice had increased manganese in the liver and reduced manganese in the bile when compared with the empty-AAV-injected control animals [11]. Additionally, immunofluorescence results depicted the localization of ZIP8 to the apical membrane of hepatocytes and the epithelial cells of the bile duct [11,12]. Together, these previous findings indicate that hepatic ZIP8 functions at the apical canalicular membrane of hepatocytes to reclaim manganese from the bile, establishing a unique role for ZIP8 in the regulation of manganese metabolism. Despite the functional significance of ZIP8, our knowledge about the regulation of ZIP8 by manganese remains limited.

Manganese is primarily acquired through diet in humans [13]. Reasonably, both manganese-deficient and manganese-overload diets have the potential to alter manganese concentrations from homeostatic levels. However, cases of manganese deficiency caused by inadequate dietary intake are extremely rare [14]. While the Institute of Medicine’s recommended adequate intakes for manganese are 2.3 mg/day and 1.8 mg/day for adult men and women, respectively [15], these requirements are easily met with the consumption of regular diets since a wide range of foods can provide sufficient manganese. As opposed to the rare occurrence of diet-related manganese deficiency [14], manganese toxicity poses a more prevalent health concern due to widespread environmental overexposure. Manganese overload is a significant health concern among professionals with a high risk of inhaling excess manganese [16,17], for individuals who possess one or more specific genotypes associated with disrupted manganese metabolism [18,19], and for populations that live in areas with manganese-contaminated food or water [20,21]. A significant knowledge gap regarding how increased manganese intake may affect ZIP8 expression remains. Therefore, to better understand the regulation of ZIP8 in high-manganese conditions, this present study aimed to use mice as a model system to examine the responses of ZIP8’s protein expression to a high-manganese diet.

## 2. Results

### 2.1. ZIP8 Expression in the Mouse Lung, Kidney, Spleen, Liver, and Heart

**Figure 1:**
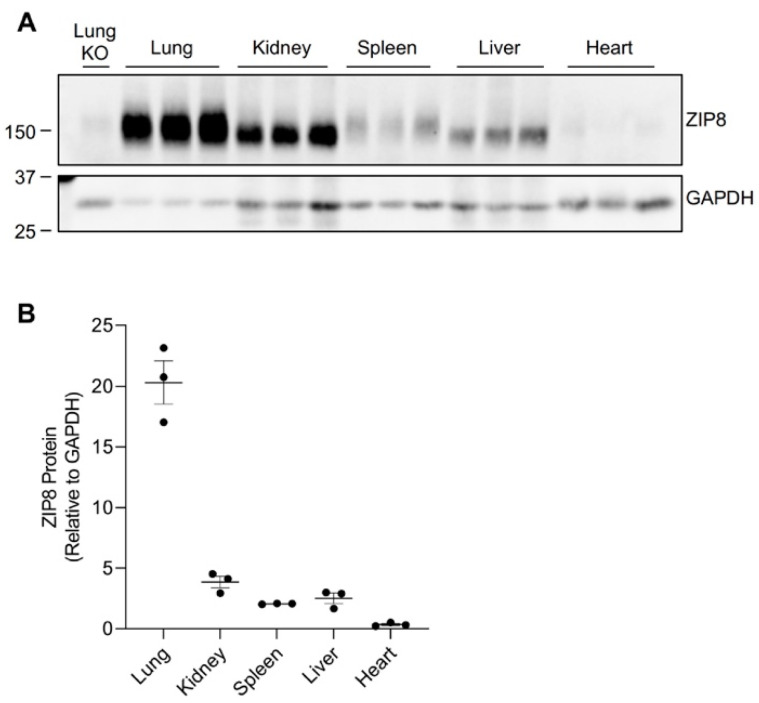
ZIP8 expression in the lung, kidney, spleen, liver, and heart of mice. Mouse tissues were collected from wild-type male mice at 3 weeks of age (*n* = 3). (**A**) Tissues were analyzed by Western blotting with 120 μg protein loaded per lane. The blot was probed with anti-mouse ZIP8 antibody for ZIP8 detection and HRP-conjugated GAPDH antibody as the loading control. The blot includes the lung tissue from a female, 6-week-old *Zip8*-KO mouse as a control to validate the anti-mouse ZIP8 antibody. (**B**) For each tissue, the ZIP8 protein level was normalized to GAPDH.

Previous human and animal studies have found that ZIP8 is ubiquitously expressed throughout the body [4,22,23,24]. Transcriptome analyses of normal mouse tissues revealed an abundant presence of ZIP8 mRNA in the lung, kidney, spleen, liver, and heart [23]. To determine the pattern of ZIP8 protein expression in these five organs, we collected tissues from age- and sex-matched mice fed a traditional rodent diet and assessed ZIP8 levels by Western blot analysis. To verify the specificity of the anti-mouse ZIP8 antibody, we used the lung tissue collected from tamoxifen-inducible *Zip8*-knockout (*Zip8*-KO) mice. We first verified that the anti-mouse ZIP8 antibody detects a ZIP8-specific band at ~150 kDa (Figure 1A and Figure S1). We then compared ZIP8 protein levels and found that ZIP8 can be detected in all five organs, with the lung exhibiting the highest and the heart exhibiting the lowest levels of ZIP8 expression (Figure 1A,B). 

### 2.2. High-Manganese Diet Does Not Change ZIP8 Expression in the Lung, Kidney, and Spleen of Young Mice

Previous studies indicated that human infants demonstrate a higher absorption rate of ingested manganese and, therefore, an increased sensitivity to manganese compared with adults [25,26,27]. Exposing a child to high levels of manganese during the prenatal and early postnatal periods can negatively impact neurodevelopment [28,29], and these adverse effects may be facilitated by an infant’s increased susceptibility to manganese intoxication. Infant and child populations at high risk of manganese hyperaccumulation include pediatric patients that receive total parenteral nutrition (TPN), which is a commonly used procedure to nourish critically ill patients [30]. Studying animals that were supplied with a high-manganese dietary source during the neonatal period can lead to a better understanding of the role of ZIP8 in manganese metabolism at the early stages of development. In this study, we utilized mice from our neonatal manganese overload model to investigate the regulation of ZIP8 by high-manganese intake in young animals. 

**Figure 2:**
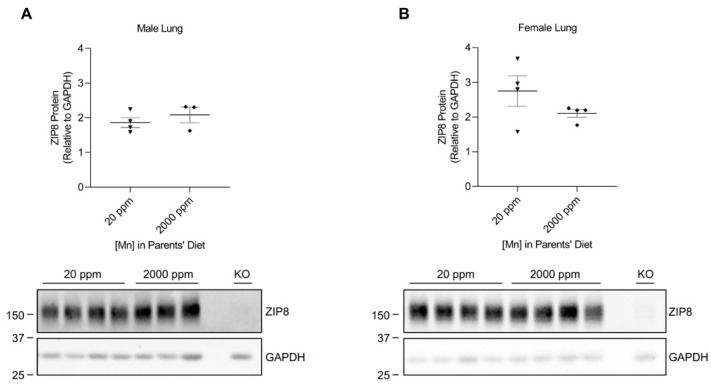
Comparison of ZIP8 protein expressions in the lung between the control and high-manganese diet groups. Lung samples were collected from pups at 3 weeks of age. Expression of ZIP8 in male (**A**) and female (**B**) pups that were born from and fed by mothers on 20 ppm manganese and 2000 ppm manganese diets were analyzed by Western blot analyses (*n* = 3–4/group). Equal amounts of protein (50 μg for male lung samples and 102 μg for female lung samples) were assessed. Blots include the lung sample from a female, 6-week-old *Zip8*-KO mouse (fed a traditional rodent diet) as a control to validate the anti-mouse ZIP8 antibody. Quantification of ZIP8 levels were determined using GAPDH as the loading control. Data are expressed as mean ± SEM.

**Figure 3:**
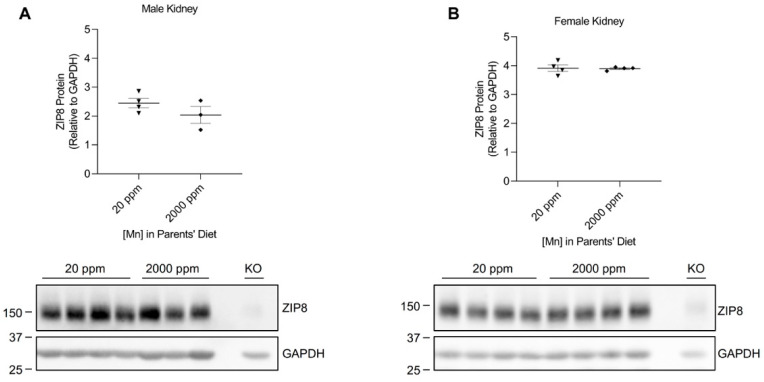
Comparison of ZIP8 levels in the kidney between the control and high-manganese diet groups. Kidney samples were collected from pups at 3 weeks of age for Western blot analysis. Expression of ZIP8 in male (**A**) and female (**B**) pups that were born from and fed by mothers on 20 ppm manganese and 2000 ppm manganese diets were compared (*n* = 3–4/group). Samples with equal amounts of protein (159 μg) were analyzed. Blots include the kidney sample from a female, 6-week-old *Zip8*-KO mouse (fed a traditional rodent diet) as a control. Quantification of ZIP8 levels were determined using GAPDH as the loading control. Data are expressed as mean ± SEM.

**Figure 4:**
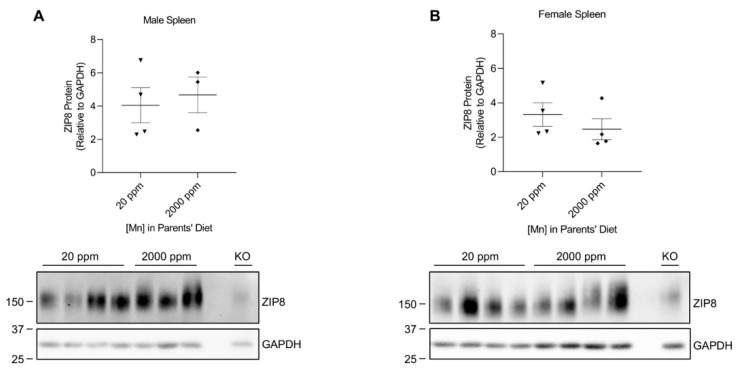
Comparison of spleen ZIP8 levels between the control and high-manganese diet groups. Spleen samples were collected from pups at 3 weeks of age. ZIP8 in male (**A**) and female (**B**) pups that were born from and fed by mothers on 20 ppm manganese and 2000 ppm manganese diets were analyzed by Western blot analyses (*n* = 3–4/group). Samples with equal amounts of protein (124 μg) were assessed. Blots include the spleen sample from a female, 6-week-old *Zip8*-KO mouse (fed a traditional rodent diet) as a control. Quantification of ZIP8 levels were determined using GAPDH as the loading control. Data are expressed as mean ± SEM.

We developed our neonatal manganese overload model by feeding breeder mice a purified diet containing 20 ppm (control) or 2000 ppm (high) of manganese throughout mating, gestation, and lactation. Our previous study determined that the manganese content in the maternal milk from mice on the high-manganese diet was about 3 times that from mice on the control diet [31]. Given that this dietary intervention led to an alteration in breast milk manganese content, the pups experienced differences in manganese intake before weaning. Pups were sacrificed and tissue samples were collected at 3 weeks old. ZIP8 expressions were analyzed by Western blot. We first compared ZIP8 expressions in the lung, kidney, and spleen because these three organs have relatively high levels of ZIP8 protein (Figure 1). Our results indicated that high-manganese diet did not significantly affect ZIP8 protein expression in the lung (Figure 2A,B and Figure S2) or kidney (Figure 3A,B and Figure S3), despite ZIP8 bearing a crucial role in importing manganese into these organs [32,33,34]. Our results also displayed that the high-manganese diet did not significantly alter ZIP8 protein levels in the spleen (Figure 4A,B and Figure S4). Overall, our findings depicted that ZIP8 expression in the lung, kidney, and spleen of young mice is relatively high under normal conditions and is unaffected by increased manganese intake. 

### 2.3. High-Manganese Diet Leads to a Downregulation of Hepatic ZIP8 in Young Mice but Not in Adult Mice

**Figure 5:**
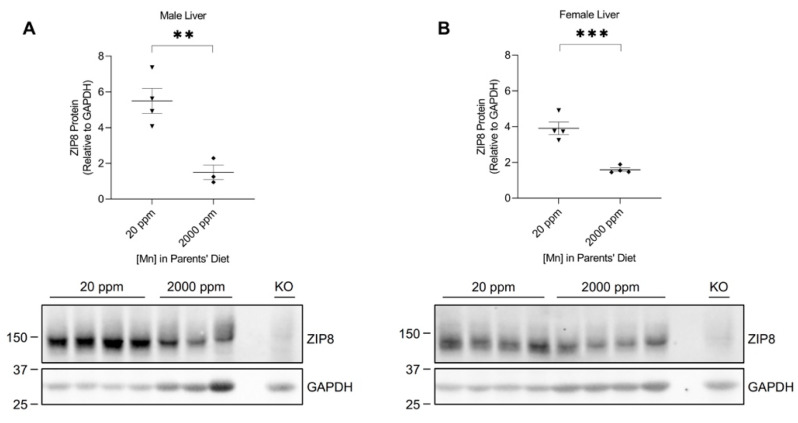
Comparison of liver ZIP8 levels between the control and high-manganese diet groups. Expressions of ZIP8 in male (**A**) and female (**B**) pups that were born from and fed by mothers on 20 ppm manganese and 2000 ppm manganese diets were compared (*n* = 3–4/group). Liver samples were collected from pups at 3 weeks of age for Western blot analysis. Equal amounts of protein (213 μg for male liver samples and 240 μg for female liver samples) were loaded per lane. Blots include the liver sample from a female, 6-week-old *Zip8*-KO mouse (fed a traditional rodent diet) as a control. Quantification of ZIP8 levels were determined using GAPDH as the loading control. Data are expressed as mean ± SEM. ** *p* < 0.01, *** *p* < 0.001.

The liver plays a crucial role in regulating manganese homeostasis by eliminating the manganese from the blood and secreting it as bile conjugate for intestinal reabsorption or fecal excretion [35,36,37]. Although ZIP8 is not highly expressed in the liver (only about 12% of the level detected in the lung when equal amounts of protein from both tissues were compared) (Figure 1), hepatic ZIP8 is indispensable for the regulation of systemic manganese homeostasis because a lack of ZIP8 in the liver leads to systemic manganese deficiency [11]. Western blot analysis of the liver samples from the neonatal model revealed that high-manganese intake reduced the expression of hepatic ZIP8 in both male (by 73%, *p* < 0.01) and female (by 59%, *p* < 0.001) mice (Figure 5A,B and Figure S5).

**Figure 6:**
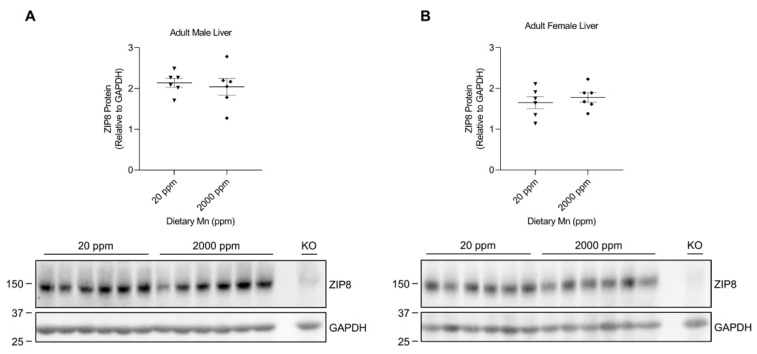
Comparison of liver ZIP8 levels between the control and high-manganese diet groups in adult mice. Expressions of liver ZIP8 in male (**A**) and female (**B**) mice on 20 ppm and 2000 ppm manganese diets were analyzed by Western blotting (*n* = 6/group). Liver samples were collected at 12 weeks of age. Equal amounts of protein (222 μg for male liver samples and 220 μg for female liver samples) were assessed. Blots include the liver sample from a female, 6-week-old *Zip8*-KO mouse (fed a traditional rodent diet) as a control. Quantification of ZIP8 levels were determined using GAPDH as the loading control. Data are expressed as mean ± SEM.

The observed differences in hepatic ZIP8 expression between the diet groups of 3-week-old mice pups prompted us to investigate whether adult mice would respond similarly when experiencing elevated manganese consumption. We previously developed an adult mouse model with a high-manganese intake [38]. Western blot analysis of the liver samples collected from the control and high-manganese intake groups revealed no significant difference in ZIP8 between these 2 groups for both sexes (Figure 6A,B and Figure S6), demonstrating that hepatic ZIP8 regulation by increased manganese intake in 12-week-old mice is different from that of 3-week-old mice.

### 2.4. Hepatic ZIP8 Expression Varies by Age in Mice

**Figure 7:**
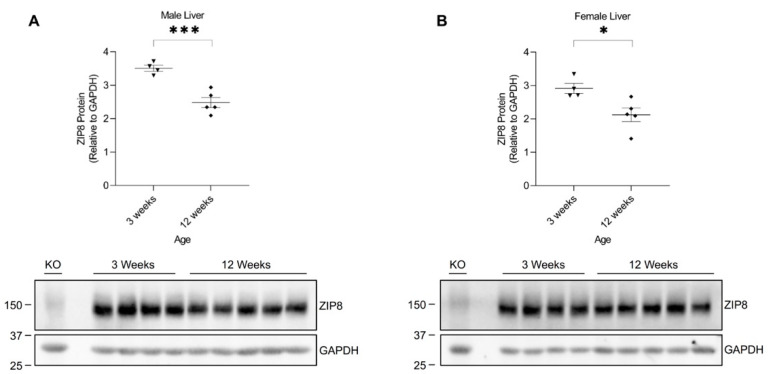
Comparison of liver ZIP8 protein levels between 3-week-old and 12-week-old mice. Expressions of ZIP8 in male (**A**) and female (**B**) mice at 3 weeks and 12 weeks of age were analyzed by Western blotting (*n* = 4–5/group). Samples with equal amounts of protein (200 μg) were assessed. Blots include the liver sample from a female, 6-week-old *Zip8*-KO mouse (fed a traditional rodent diet) as a control. Quantification of ZIP8 levels were determined using GAPDH as the loading control. Data are expressed as mean ± SEM. * *p* < 0.05, *** *p* < 0.001.

The age-associated differences in murine manganese metabolism were previously reported, showing that 3-week-old mice appeared to have higher liver manganese concentrations than 12-week-old mice [31]. Our results have shown that hepatic ZIP8 is downregulated in mice exposed to high manganese during the neonatal period (in 3-week-old mice), but not in mice receiving a high-manganese diet after reaching maturity. These results suggest an age-dependent response of hepatic ZIP8 to manganese exposure while also indicating that variations pertaining to the protein-mediated transport of manganese exist across younger and adult mice. Therefore, we sought to investigate the varying responses to a high-manganese diet produced by the young and adult mice and examine whether hepatic ZIP8 expression differs between 3-week-old and 12-week-old mice under typical dietary conditions. We collected liver tissues from 3-week-old and 12-week-old wild-type mice fed a traditional rodent diet. Western blot analysis of the tissue demonstrated that liver ZIP8 levels of the 12-week-old mice were significantly lower than that of the 3-week-old mice in both male (by 27%, *p* < 0.001) and female (by 29%, *p* < 0.05) (Figure 7A,B and Figure S7). This variation of hepatic ZIP8 expression at different developmental stages reveals a novel mechanism for the age-dependent changes in liver manganese concentrations observed previously [31]. As ZIP8 is an apical membrane manganese importer in hepatocytes and bile duct epithelial cells [11,12], the higher hepatic ZIP8 levels in 3-week-old mice correlate with a higher influx of biliary manganese into the liver and, thus, a greater hepatic manganese content. In contrast, the lower hepatic ZIP8 levels in 12-week-old mice relates to a reduced recovery of biliary manganese and, thus, a lower liver manganese content.

Additionally, the age-dependent hepatic ZIP8 expression offers a feasible explanation for the variations observed between our neonatal and adult mouse models. As ZIP8 expression is naturally higher in 3-week-old mice, these young mice can adapt to changes in the diet through downregulating ZIP8 expression when exposed to a high-manganese diet. As a result, decreasing hepatic ZIP8 increases overall biliary excretion of manganese and prevents manganese overaccumulation in the body. Contrastingly, adult mice naturally have a significantly reduced hepatic ZIP8 expression after reaching maturity. This low expression at baseline may have limited the potential for ZIP8 to be further downregulated by diet changes and, therefore, caused no significant differences in liver ZIP8 levels when exposed to a high-manganese diet.

## 3. Discussion

Manganese is a micronutrient required for enabling the metabolic activities of various enzymes [2,13,36,39]. Dietary intake is the major source of this nutrient in humans [13]. Following manganese consumption, the body utilizes absorptive and secretory mechanisms of the intestine and liver to maintain manganese homeostasis [1,2,3,37]. The hepatobiliary excretion of manganese involves a newly identified manganese importer, ZIP8. Specifically, ZIP8 functions at the apical membrane of hepatocytes to reabsorb manganese from the bile [11,12]. The physiological significance of ZIP8 has been demonstrated in both humans and animal models, where loss of ZIP8 caused severe manganese deficiency through increased biliary manganese secretion [7,8,11,12,40]. Although there is a well-recognized connection between ZIP8 and manganese metabolism, little is known about the regulation of ZIP8 by high-manganese intake. 

In the present study, we examined the regulation of ZIP8 using both neonatal and adult mouse models of dietary manganese overload. By comparing the ZIP8 expression between the control and high-manganese diet groups in the neonatal model, we discovered that high-manganese intake during the neonatal stage significantly lowered hepatic ZIP8 protein levels (Figure 5). The downregulation of hepatic ZIP8 can reduce the recovery of manganese at the canalicular membrane and, in turn, decrease hepatocyte manganese accumulation and lessen the body’s manganese burden [11]. We also found that although ZIP8 expression in the lung, kidney, and spleen was higher than that in the liver (Figure 1), the levels of ZIP8 in these three organs did not change with the increase in manganese intake (Figure 2, Figure 3 and Figure 4). Together, these findings indicate an adaptive role for hepatic ZIP8 to balance manganese homeostasis under conditions of increased manganese intake. 

Interestingly, analysis of animal livers from the adult model of manganese overload revealed that high-manganese intake during adulthood did not alter hepatic ZIP8 (Figure 6). Metal level measurement indicated that the neonatal mice with high-manganese intake had a liver manganese about three times as high as the level of control animals (Figure S8); while our previous study has demonstrated that adult mice fed a high-manganese diet had a liver manganese concentration about four times as high as the value of mice fed with the control diet [38]. The unparalleled responses to a high-manganese intake across young and adult mice suggest that mechanisms underlying the regulation of ZIP8 by dietary manganese varies between age groups. Indeed, our further analyses depicted that hepatic ZIP8 levels were significantly higher in 3-week-old mice compared with 12-week-old mice (Figure 7). This age dependence of ZIP8 expression provides one possible reason for the differences observed between the neonatal and adult mice models. As ZIP8 expression is relatively high in 3-week-old mice, these young mice can respond to the increase in diet manganese by downregulating hepatic ZIP8, which in turn will increase overall biliary manganese excretion to prevent manganese overaccumulation in the body. However, for 12-week-old adult mice, the increase in dietary manganese does not induce any changes of hepatic ZIP8 because they naturally have a lower level of ZIP8 in the liver. This absence of transporter downregulation in adult mice offers one potential explanation for their liver manganese overaccumulation when exposed to a high-manganese diet [38]. Moreover, the finding of age-dependent hepatic ZIP8 expression helps to explain the variation in the liver manganese content across mice of different ages [31]. The greater liver manganese content exhibited by 3-week-old mice [31] can be explained by a higher hepatic ZIP8 level and, thus, a higher influx of biliary manganese into the liver. In contrast, the lower liver manganese concentrations in 12-week-old mice [31] can be attributed to the lower hepatic ZIP8 level and, hence, a reduced recovery of biliary manganese. While our animal models showed a decrease in manganese retention in later stages of development, this age-related variation in manganese metabolism was also seen in humans where adults were reported to have lower retention of ingested manganese compared with children and infants [25,26,27]. Therefore, findings from the present study may benefit future research that aims to further investigate mechanisms of the age-associated differences in manganese metabolism. Moreover, future studies are required to determine the exact mechanism through which high manganese affects ZIP8 protein expression in neonatal mice. 

In conclusion, our study discovered that hepatic ZIP8 in young mice decreases in response to a high-manganese intake. We also found that younger mice exhibit higher hepatic ZIP8 levels compared with adult mice, illustrating that hepatic ZIP8 expression varies based on age and provides an explanation for the age-dependent variation in liver manganese concentrations. Together, these results offer novel insights into ZIP8’s function in regulating manganese metabolism.

## 4. Materials and Methods

### 4.1. Animals, Genotyping, and Tissue Collection

All procedures for animal experiments were approved by the Institutional Animal Care and Use Committees of the University of Arizona. Animal cages containing 5 or fewer mice were kept at 21–22 °C with a 12 h light/dark cycle. All mice were provided with tap water ad libitum. Under normal dietary conditions, mice were weaned at 3 weeks of age and fed a traditional rodent diet (Teklad 7913; Envigo, Indianapolis, IN, USA). Inducible-*Zip8* knockout mice were generated as previously described [41]. Animal genotypes were identified using a Mouse Direct PCR kit (Bimake, Houston, TX, USA) and the following primers. For *Zip8*^flox/flox^ mice: 2476_27, 5′-CAG GGT TTC TCT GTG TAA CAG G-3′; 2474_28, 5′-AGT GTA CAG GCT CCA GCT ACC-3′. For Ubc-Cre mice: 25285, 5′-GAC GTC ACC CGT TCT GTT G-3′; oIMR7338, 5′-CTA GGC CAC AGA ATT GAA AGA TCT-3′; oIMR7339, 5′-GTA GGT GGA AAT TCT AGC ATC ATC C-3′; oIMR9074, 5′-AGG CAA ATT TTG GTG TAC GG-3′. The knockout of ZIP8 protein in *Zip8*^flox/flox, UBC-CRE^ mice was further confirmed using Western blotting and anti-mouse ZIP8 antibodies that were generated as previously described [41]. All mice in this study were sacrificed after injection of ketamine/xylazine anesthesia. Following tissue collection, samples were immediately frozen in liquid nitrogen and stored at −80 °C until further use.

### 4.2. Mouse Models with High-Manganese Intake

**Figure 8:**
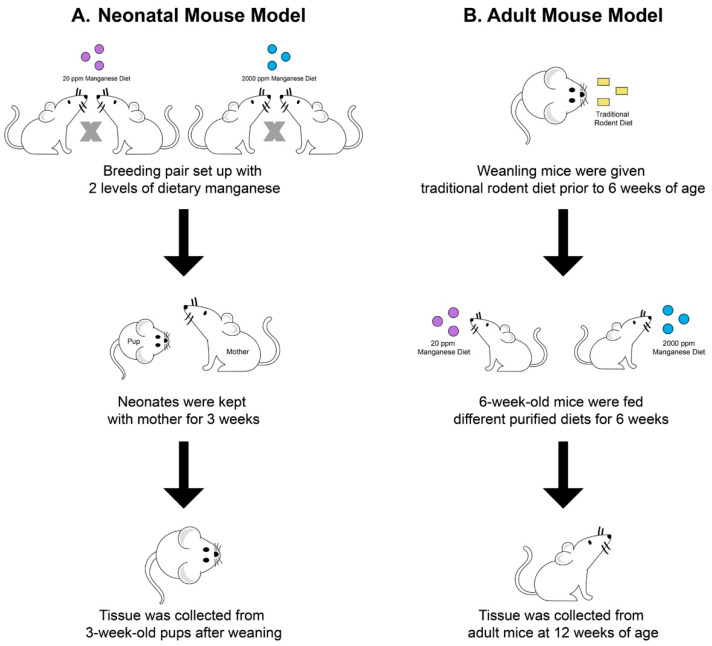
Schematic illustration of experimental procedures for establishing the two animal models. In the neonatal manganese overload model (**A**), 8–9-week old mating mice were fed diets containing 20 ppm or 2000 ppm manganese throughout breeding and lactation. Mice pups were sacrificed at 3 weeks old after weaning. In the adult manganese overload model (**B**), 6-week-old mice transitioned from receiving a traditional rodent diet to a purified diet containing 20 ppm or 2000 ppm manganese. After receiving purified diets for 6 weeks, mice were sacrificed at 12 weeks old.

The creation of the manganese overload conditions in young mice and adult mice was described previously [31,38]. Briefly, for the neonatal manganese overload model, mating mice were fed with a modified AIN-93G diet containing 20 ppm or 2000 ppm manganese starting at 8–9 weeks of age. Mating mice consumed these diets throughout breeding and lactation periods for a total of 6–7 weeks. The progenies of the mating mice were fed a maternal milk diet from birth to 3 weeks of age. Tissues were harvested from the progenies at the weaning age of 21 days (Figure 8A). For the adult manganese overload model, weanling (21-day-old) wild-type mice were given a traditional rodent diet between 3 weeks and 6 weeks of age. Starting at 6 weeks of age, the mice transitioned to receiving a modified AIN-93G animal diet containing 20 ppm or 2000 ppm of manganese (Figure 8B). The mice were euthanized at 12 weeks of age for tissue collection.

### 4.3. Western Blot Analysis

Mouse tissues were homogenized in ice-cold NETT lysis buffer (150 mM NaCl + 5 mM EDTA + 10 mM Tris + 1% Triton X-100 in deionized water + 1x Protease inhibitor (Bimake, Houston, TX, USA)). Protein concentrations of tissue homogenates were determined in duplicates using RC DC Protein Assay (Bio-Rad Life Science, Hercules, CA, USA). Samples with equal amounts of protein were mixed with 1x Laemmli buffer, and then incubated at 37 °C for 30 min. Proteins were electrophoretically separated on 10% sodium dodecyl sulfate polyacrylamide gel and transferred to nitrocellulose membrane (GVS, Sanford, ME, USA). Membranes were blocked with blocking buffer (5% non-fat dried milk in TBST (tris-buffered saline + 0.1% Tween 20)) for 1–3 h at room temperature and incubated with rabbit anti-mouse ZIP8 antibody (1:1000) at 4 °C overnight. Following 4 washes with TBST (5 min per wash), membranes were incubated with horseradish peroxidase (HRP)-conjugated anti-rabbit secondary antibody (1:3000, GE Healthcare, Chicago, IL, USA). Prior to imaging, membranes were washed twice with TBST and then twice with TBS (5 min per wash). The signal was detected using an enhanced chemiluminescent substrate (SuperSignal West Pico, Thermo Fisher Scientific, Waltham, MA, USA) and ChemiDoc MP Imaging System (Bio-Rad Life Science, Hercules, CA, USA). For the gel loading control, membranes were probed with an HRP-conjugated anti-GAPDH antibody (1:6000, Proteintech, Rosemont, IL, USA). 

### 4.4. Statistical Analysis

Data were analyzed using GraphPad Prism (GraphPad Prism 8 Software, San Diego, CA, USA). Equal variances between groups were assessed using the *F*-test, and between-group comparisons of ZIP8 levels were performed using the unpaired Student’s *t*-test. A *p*-value < 0.05 was accepted as statistically significant.

## Data Availability

The data presented in this study are available on request from the corresponding author.

## Supplementary Materials

The following supporting information can be downloaded at: https://www.mdpi.com/article/10.3390/ijms24065962/s1.

## References

[1] Madejczyk M.S., Boyer J.L., Ballatori N. (2009). Hepatic uptake and biliary excretion of manganese in the little skate, Leucoraja erinacea. Comparative Biochem. Physiol. C Toxicol. Pharmacol..

[2] Horning K.J., Caito S.W., Tipps K.G., Bowman A.B., Aschner M. (2015). Manganese Is Essential for Neuronal Health. Annu. Rev. Nutr..

[3] Teeguarden J.G., Dorman D.C., Covington T.R., Clewell H.J., Andersen M.E. (2007). Pharmacokinetic modeling of manganese. I. Dose dependencies of uptake and elimination. J. Toxicol. Environ. Health A.

[4] Nebert D.W., Liu Z. (2019). SLC39A8 gene encoding a metal ion transporter: Discovery and bench to bedside. Hum. Genom..

[5] Wang C.Y., Jenkitkasemwong S., Duarte S., Sparkman B.K., Shawki A., Mackenzie B., Knutson M.D. (2012). ZIP8 is an iron and zinc transporter whose cell-surface expression is up-regulated by cellular iron loading. J. Biol. Chem..

[6] Choi E.K., Nguyen T.T., Gupta N., Iwase S., Seo Y.A. (2018). Functional analysis of SLC39A8 mutations and their implications for manganese deficiency and mitochondrial disorders. Sci. Rep..

[7] Boycott K.M., Beaulieu C.L., Kernohan K.D., Gebril O.H., Mhanni A., Chudley A.E., Redl D., Qin W., Hampson S., Kury S. (2015). Autosomal-Recessive Intellectual Disability with Cerebellar Atrophy Syndrome Caused by Mutation of the Manganese and Zinc Transporter Gene SLC39A8. Am. J. Hum. Genet..

[8] Riley L.G., Cowley M.J., Gayevskiy V., Roscioli T., Thorburn D.R., Prelog K., Bahlo M., Sue C.M., Balasubramaniam S., Christodoulou J. (2017). A SLC39A8 variant causes manganese deficiency, and glycosylation and mitochondrial disorders. J. Inherit. Metab. Dis..

[9] Park J.H., Hogrebe M., Gruneberg M., DuChesne I., von der Heiden A.L., Reunert J., Schlingmann K.P., Boycott K.M., Beaulieu C.L., Mhanni A.A. (2015). SLC39A8 Deficiency: A Disorder of Manganese Transport and Glycosylation. Am. J. Hum. Genet..

[10] Galvez-Peralta M., He L., Jorge-Nebert L.F., Wang B., Miller M.L., Eppert B.L., Afton S., Nebert D.W. (2012). ZIP8 zinc transporter: Indispensable role for both multiple-organ organogenesis and hematopoiesis in utero. PLoS ONE.

[11] Lin W., Vann D.R., Doulias P.T., Wang T., Landesberg G., Li X., Ricciotti E., Scalia R., He M., Hand N.J. (2017). Hepatic metal ion transporter ZIP8 regulates manganese homeostasis and manganese-dependent enzyme activity. J. Clin. Investig..

[12] Sunuwar L., Frkatovic A., Sharapov S., Wang Q., Neu H.M., Wu X., Haritunians T., Wan F., Michel S., Wu S. (2020). Pleiotropic ZIP8 A391T implicates abnormal manganese homeostasis in complex human disease. JCI Insight.

[13] Aschner J.L., Aschner M. (2005). Nutritional aspects of manganese homeostasis. Mol. Asp. Med..

[14] Aschner M., Erikson K. (2017). Manganese. Adv. Nutr..

[15] Institute of Medicine (US) Panel on Micronutrients (2001). Dietary Reference Intakes for Vitamin A, Vitamin K, Arsenic, Boron, Chromium, Copper, Iodine, Iron, Manganese, Molybdenum, Nickel, Silicon, Vanadium, and Zinc.

[16] Kulshreshtha D., Ganguly J., Jog M. (2021). Manganese and Movement Disorders: A Review. J. Mov. Disord..

[17] Bowler R.M., Gocheva V., Harris M., Ngo L., Abdelouahab N., Wilkinson J., Doty R.L., Park R., Roels H.A. (2011). Prospective study on neurotoxic effects in manganese-exposed bridge construction welders. Neurotoxicology.

[18] Tuschl K., Meyer E., Valdivia L.E., Zhao N., Dadswell C., Abdul-Sada A., Hung C.Y., Simpson M.A., Chong W.K., Jacques T.S. (2016). Mutations in SLC39A14 disrupt manganese homeostasis and cause childhood-onset parkinsonism-dystonia. Nat. Commun..

[19] Tuschl K., Clayton P.T., Gospe S.M., Gulab S., Ibrahim S., Singhi P., Aulakh R., Ribeiro R.T., Barsottini O.G., Zaki M.S. (2012). Syndrome of hepatic cirrhosis, dystonia, polycythemia, and hypermanganesemia caused by mutations in SLC30A10, a manganese transporter in man. Am. J. Hum. Genet..

[20] Kullar S.S., Shao K., Surette C., Foucher D., Mergler D., Cormier P., Bellinger D.C., Barbeau B., Sauve S., Bouchard M.F. (2019). A benchmark concentration analysis for manganese in drinking water and IQ deficits in children. Environ. Int..

[21] O’Neal S.L., Zheng W. (2015). Manganese Toxicity Upon Overexposure: A Decade in Review. Curr. Environ. Health Rep..

[22] Wang B., Schneider S.N., Dragin N., Girijashanker K., Dalton T.P., He L., Miller M.L., Stringer K.F., Soleimani M., Richardson D.D. (2007). Enhanced cadmium-induced testicular necrosis and renal proximal tubule damage caused by gene-dose increase in a Slc39a8-transgenic mouse line. Am. J. Physiol. Cell Physiol..

[23] Yue F., Cheng Y., Breschi A., Vierstra J., Wu W., Ryba T., Sandstrom R., Ma Z., Davis C., Pope B.D. (2014). A comparative encyclopedia of DNA elements in the mouse genome. Nature.

[24] Fagerberg L., Hallstrom B.M., Oksvold P., Kampf C., Djureinovic D., Odeberg J., Habuka M., Tahmasebpoor S., Danielsson A., Edlund K. (2014). Analysis of the human tissue-specific expression by genome-wide integration of transcriptomics and antibody-based proteomics. Mol. Cell. Proteom..

[25] Neal A.P., Guilarte T.R. (2013). Mechanisms of lead and manganese neurotoxicity. Toxicol. Res..

[26] Johnson P.E., Lykken G.I., Korynta E.D. (1991). Absorption and biological half-life in humans of intrinsic and extrinsic 54Mn tracers from foods of plant origin. J. Nutr..

[27] Dorner K., Dziadzka S., Hohn A., Sievers E., Oldigs H.D., Schulz-Lell G., Schaub J. (1989). Longitudinal manganese and copper balances in young infants and preterm infants fed on breast-milk and adapted cow’s milk formulas. Br. J. Nutr..

[28] Claus Henn B., Ettinger A.S., Schwartz J., Tellez-Rojo M.M., Lamadrid-Figueroa H., Hernandez-Avila M., Schnaas L., Amarasiriwardena C., Bellinger D.C., Hu H. (2010). Early postnatal blood manganese levels and children’s neurodevelopment. Epidemiology.

[29] Chung S.E., Cheong H.K., Ha E.H., Kim B.N., Ha M., Kim Y., Hong Y.C., Park H., Oh S.Y. (2015). Maternal Blood Manganese and Early Neurodevelopment: The Mothers and Children’s Environmental Health (MOCEH) Study. Environ. Health Perspect..

[30] Aschner J.L., Anderson A., Slaughter J.C., Aschner M., Steele S., Beller A., Mouvery A., Furlong H.M., Maitre N.L. (2015). Neuroimaging identifies increased manganese deposition in infants receiving parenteral nutrition. Am. J. Clin. Nutr..

[31] Wu Y., Wei G., Zhao N. (2021). Restriction of Manganese Intake Prevents the Onset of Brain Manganese Overload in Zip14(^−/−^) Mice. Int. J. Mol. Sci..

[32] Scheiber I.F., Alarcon N.O., Zhao N. (2019). Manganese Uptake by A549 Cells is Mediated by Both ZIP8 and ZIP14. Nutrients.

[33] Fujishiro H., Yano Y., Takada Y., Tanihara M., Himeno S. (2012). Roles of ZIP8, ZIP14, and DMT1 in transport of cadmium and manganese in mouse kidney proximal tubule cells. Metallomics.

[34] Fujishiro H., Hamao S., Isawa M., Himeno S. (2019). Segment-specific and direction-dependent transport of cadmium and manganese in immortalized S1, S2, and S3 cells derived from mouse kidney proximal tubules. J. Toxicol. Sci..

[35] Avila D.S., Puntel R.L., Aschner M. (2013). Manganese in health and disease. Met. Ions Life Sci..

[36] Winslow J.W.W., Limesand K.H., Zhao N. (2020). The Functions of ZIP8, ZIP14, and ZnT10 in the Regulation of Systemic Manganese Homeostasis. Int. J. Mol. Sci..

[37] Gurol K.C., Aschner M., Smith D.R., Mukhopadhyay S. (2022). Role of excretion in manganese homeostasis and neurotoxicity: A historical perspective. Am. J. Physiol. Gastrointest. Liver Physiol..

[38] Felber D.M., Wu Y., Zhao N. (2019). Regulation of the Metal Transporters ZIP14 and ZnT10 by Manganese Intake in Mice. Nutrients.

[39] Chen P., Bornhorst J., Aschner M. (2018). Manganese metabolism in humans. Front. Biosci..

[40] Nakata T., Creasey E.A., Kadoki M., Lin H., Selig M.K., Yao J., Lefkovith A., Daly M.J., Graham D.B., Xavier R.J. (2020). A missense variant in SLC39A8 confers risk for Crohn’s disease by disrupting manganese homeostasis and intestinal barrier integrity. Proc. Natl. Acad. Sci. USA.

[41] Wei G., Wu Y., Zhao N. (2021). Generation of a Polyclonal Antibody against the Mouse Metal Transporter ZIP8. Antibodies.

