# Supplementary material for: The Regulation of ZIP8 by Dietary Manganese in Mice
Source: Int J Mol Sci. 2023 Mar 22;24(6):5962. doi: 10.3390/ijms24065962 (PMC10056016; doi:10.3390/ijms24065962)
Supplement: Supplementary file 1 [file ijms-24-05962-s001.zip › ijms-2239700-supplementary.pdf]

The Regulation of ZIP8 by Dietary Manganese in Mice

Suetmui Yu and Ningning Zhao*

School of Nutritional Sciences and Wellness, The University of Arizona, Tucson, AZ 85721, USA
*Correspondence: zhaonn@arizona.edu; Tel.: +1-(520)-621-9744

Supplemental Figures
(Uncropped Western Blot Images for Figures S1–S7 and Figure S8)

Figure 1A ZIP8 Figure 1B GAPDH

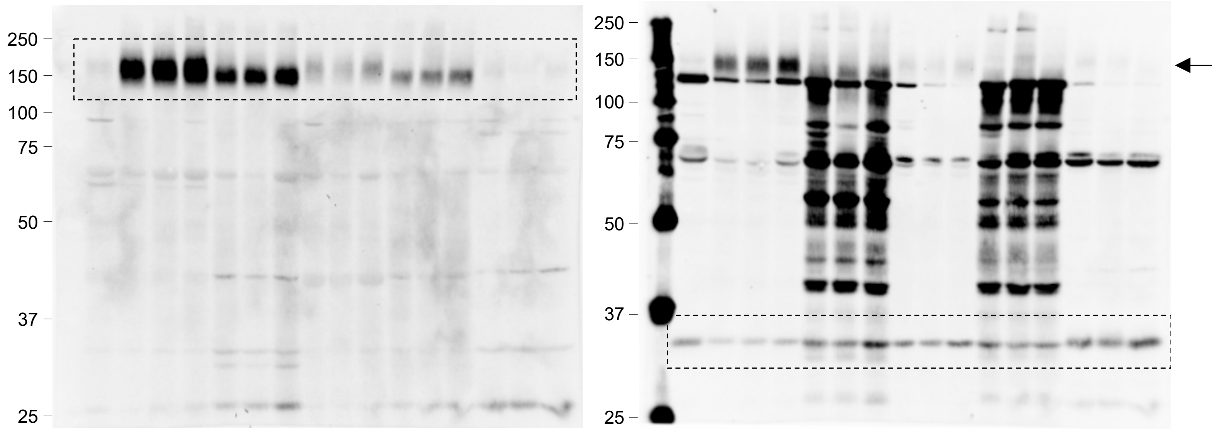

Figure S1. Uncropped Western Blot Images for Figure 1. Black arrow in the GAPDH blot indicates the unstripped signal from the anti-mZIP8 antibody. The band below 37 kDa represents the signal for GAPDH. The Precision Protein StrepTactin-HRP conjugate (Bio-Rad, #1610380) was used to detect the protein standards (Bio-Rad, #1610376) as the molecular weight markers (the lane on the very left). The StrepTactin-HRP also recognizes several non-specific bands in mouse tissue samples. Each lane was loaded with 120 μg of protein.

Figure 2A ZIP8

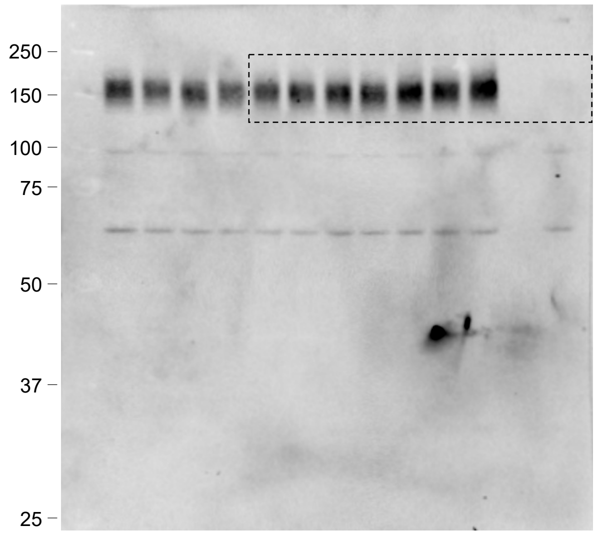

Figure 2A GAPDH

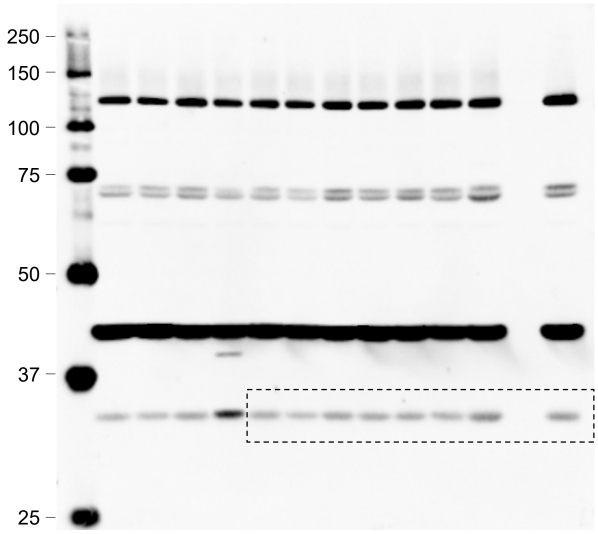

Figure 2B ZIP8

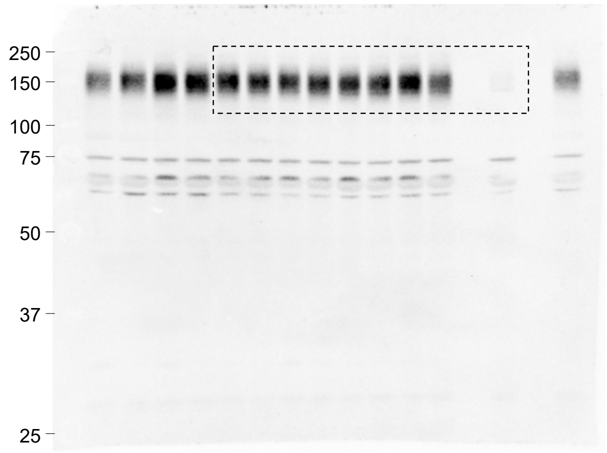

Figure 2B GAPDH

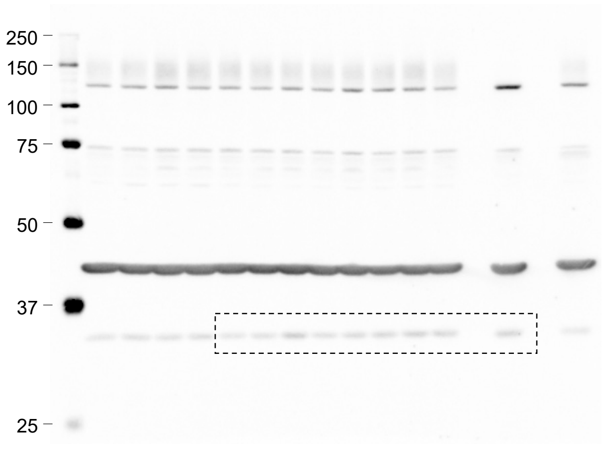

Figure S2. Uncropped Western Blot Images for Figure 2. Each lane was loaded with 50 μg of protein for Figure 2A and 102 μg of protein for Figure 2B.

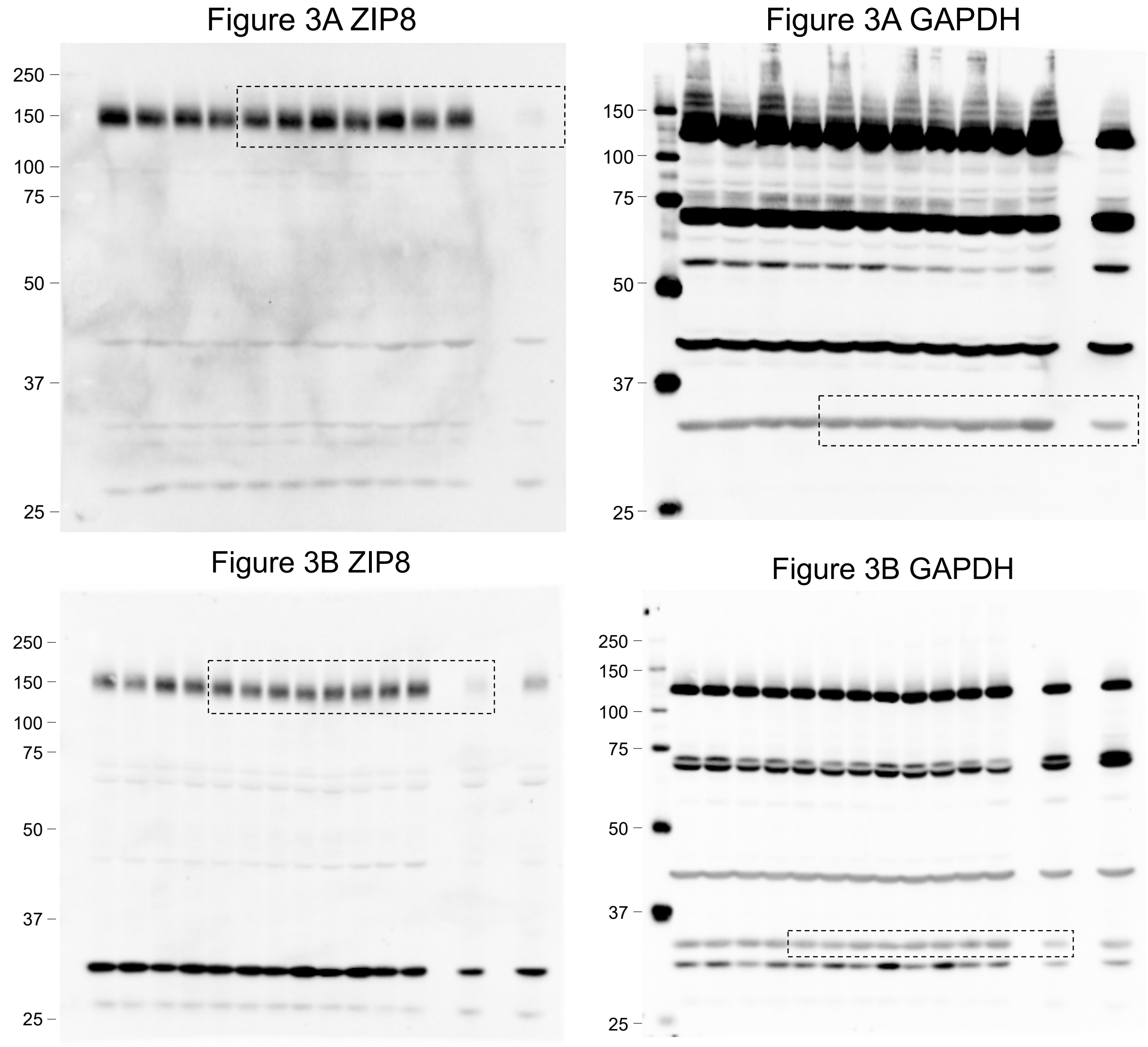

Figure S3. Uncropped Western Blot Images for Figure 3. Each lane was loaded with 159 µg of protein.

Figure 4A ZIP8

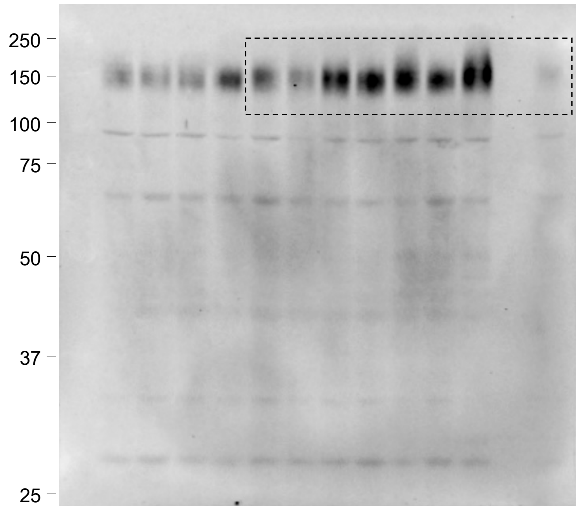

Figure 4A GAPDH

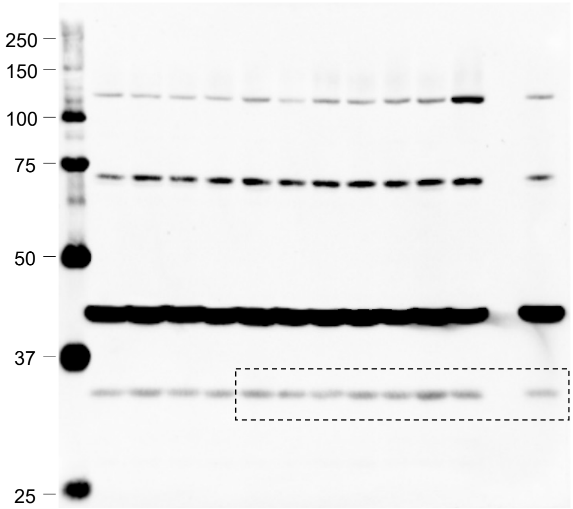

Figure 4B ZIP8

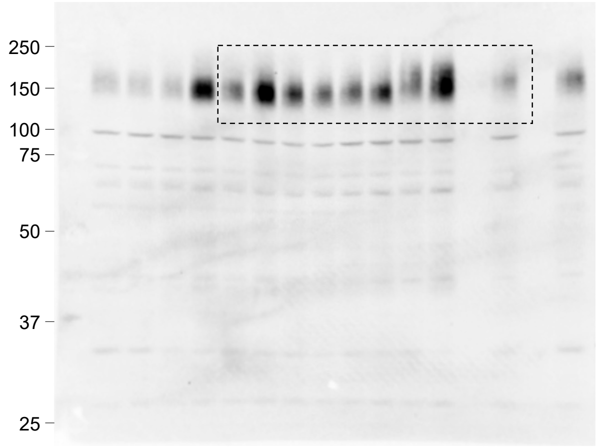

Figure 4B GAPDH

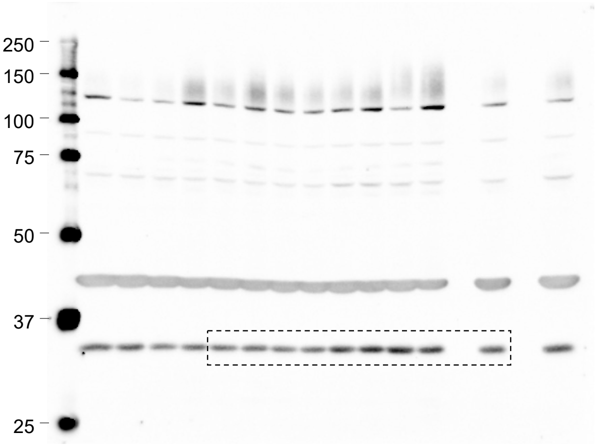

Figure S4. Uncropped Western Blot Images for Figure 4. Each lane was loaded with 124 µg of protein.

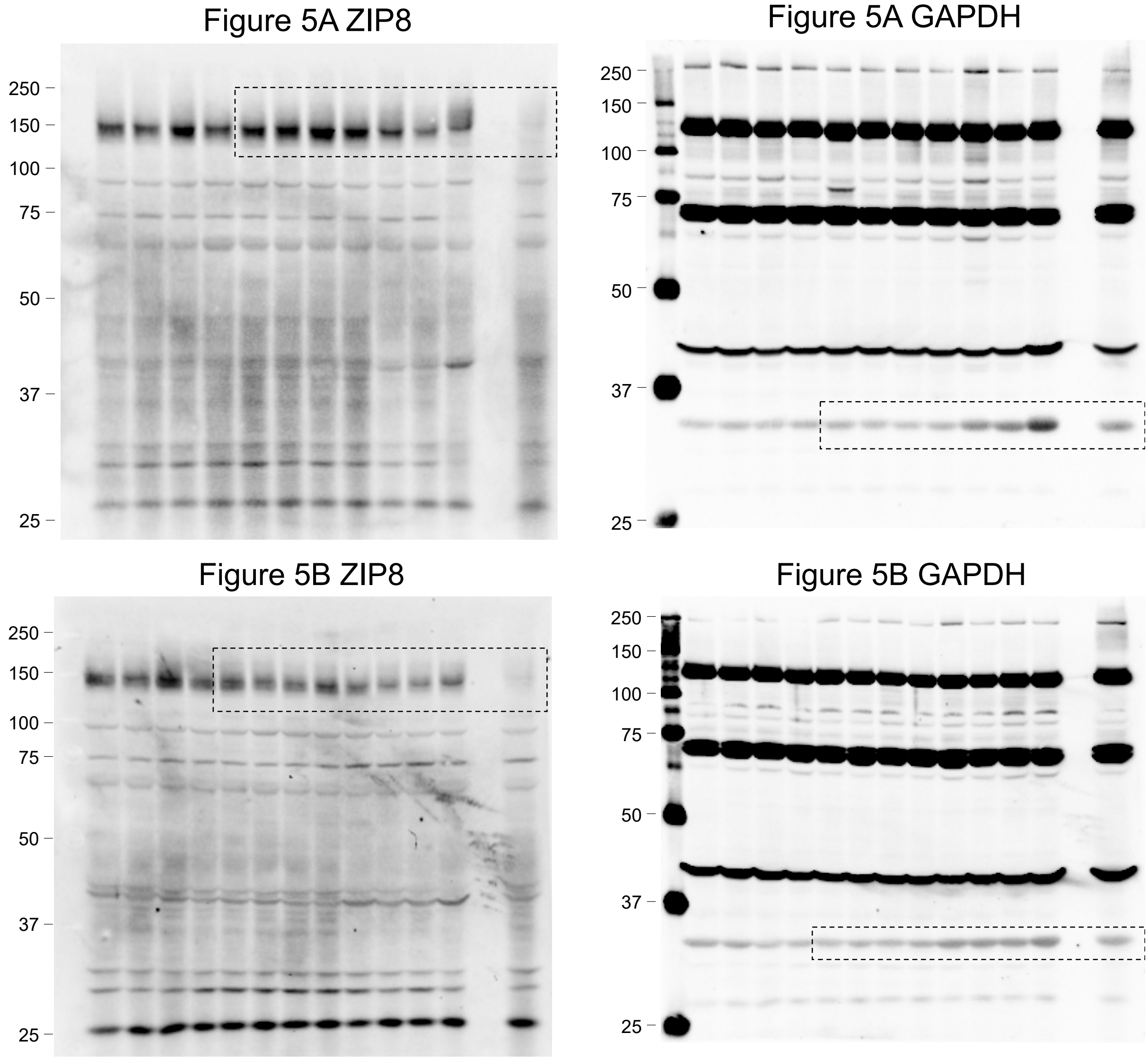

Figure S5. Uncropped Western Blot Images for Figure 5. Each lane was loaded with 213 µg of protein for Figure 5A and 240 µg of protein for Figure 5B.

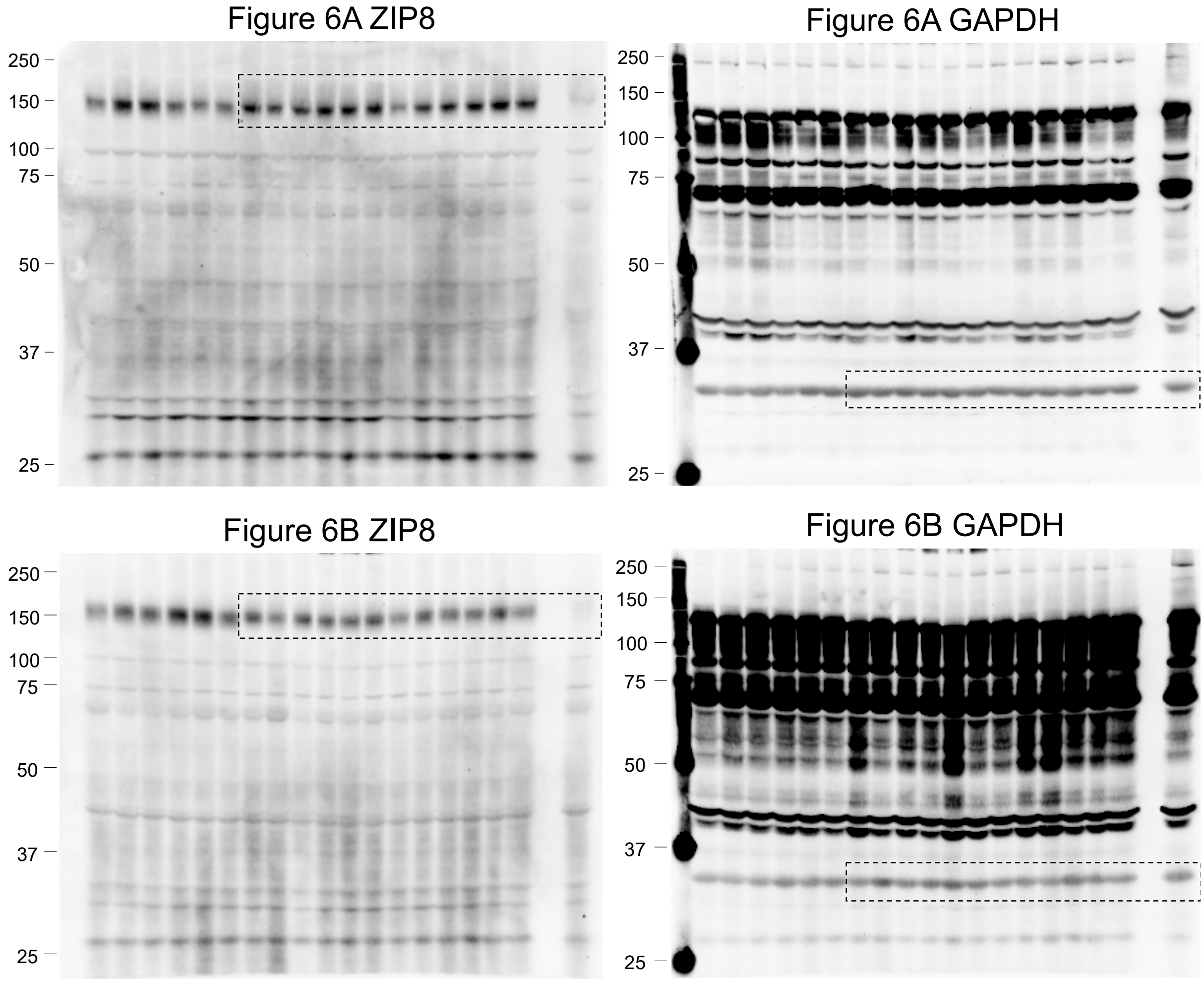

Figure S6. Uncropped Western Blot Images for Figure 6. Each lane was loaded with 222 μg of protein for Figure 6A and 220 μg of protein for Figure 6B.

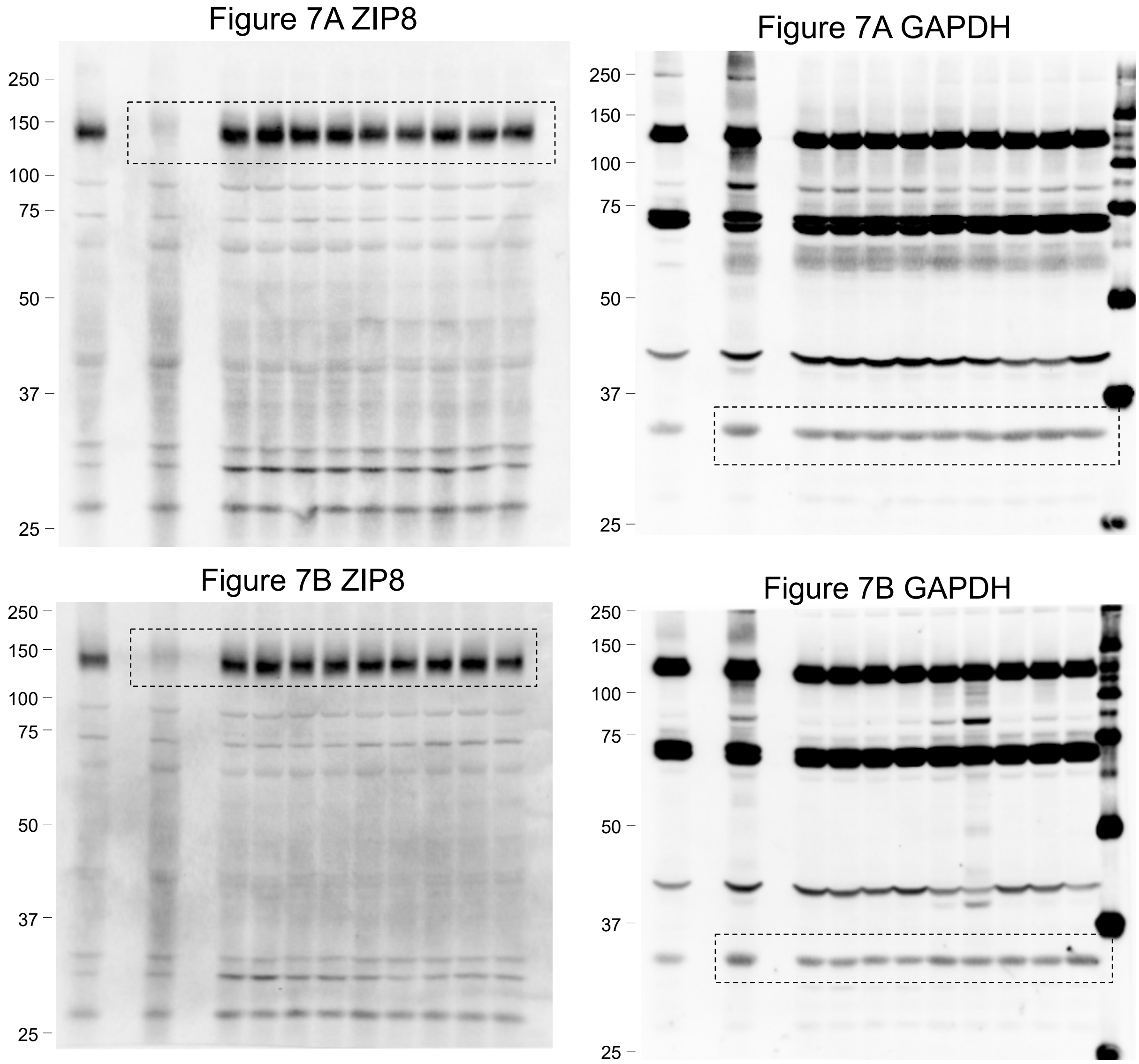

Figure S7. Uncropped Western Blot Images for Figure 7. Each lane was loaded with 200 μg of protein.

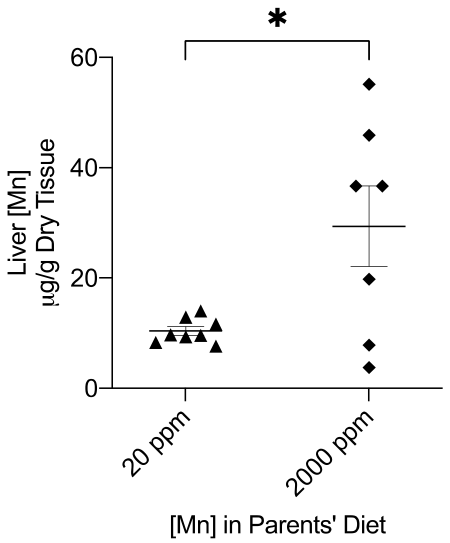

Figure S8. Liver manganese levels in mice that were born from and fed by mothers on 20 ppm manganese and 2000 ppm manganese diets. Manganese contents were measured by inductively coupled plasma mass spectrometry at 3 weeks of age (n = 7-8/group: 4 male and 4 female for the 20 ppm group; 3 male and 4 female for the 2000 ppm group). On average, the liver manganese content in mice with high manganese intake is about 3 times as high as the value from the control mice. Statistical analysis was performed using unpaired *t*-test with Welch's correction for unequal variances. Data are expressed as mean ± SEM. * $p < 0.05$.
